# A Bayesian approach for estimating allele-specific expression from RNA-Seq data with diploid genomes

**DOI:** 10.1186/s12864-015-2295-5

**Published:** 2016-01-11

**Authors:** Naoki Nariai, Kaname Kojima, Takahiro Mimori, Yosuke Kawai, Masao Nagasaki

**Affiliations:** Present address: Institute for Genomic Medicine, University of California, San Diego, 9500 Gilman Drive, La Jolla, 92093 California USA; Department of Integrative Genomics, Tohoku Medical Megabank Organization, Tohoku University, 2-1 Seiryo-machi, Aoba-ku, Sendai, Miyagi, 980-8575 Japan

**Keywords:** Allele-specific expression, RNA-Seq data, Bayesian inference

## Abstract

**Background:**

RNA-sequencing (RNA-Seq) has become a popular tool for transcriptome profiling in mammals. However, accurate estimation of allele-specific expression (ASE) based on alignments of reads to the reference genome is challenging, because it contains only one allele on a mosaic haploid genome. Even with the information of diploid genome sequences, precise alignment of reads to the correct allele is difficult because of the high-similarity between the corresponding allele sequences.

**Results:**

We propose a Bayesian approach to estimate ASE from RNA-Seq data with diploid genome sequences. In the statistical framework, the haploid choice is modeled as a hidden variable and estimated simultaneously with isoform expression levels by variational Bayesian inference. Through the simulation data analysis, we demonstrate the effectiveness of the proposed approach in terms of identifying ASE compared to the existing approach. We also show that our approach enables better quantification of isoform expression levels compared to the existing methods, TIGAR2, RSEM and Cufflinks. In the real data analysis of the human reference lymphoblastoid cell line GM12878, some autosomal genes were identified as ASE genes, and skewed paternal X-chromosome inactivation in GM12878 was identified.

**Conclusions:**

The proposed method, called ASE-TIGAR, enables accurate estimation of gene expression from RNA-Seq data in an allele-specific manner. Our results show the effectiveness of utilizing personal genomic information for accurate estimation of ASE. An implementation of our method is available at http://nagasakilab.csml.org/ase-tigar.

## Background

Allele-specific expression (ASE) has been traditionally studied in the context of genomic imprinting, in which the expression of genes depends on whether they are paternally or maternally inherited. X-chromosome inactivation is also a form of ASE, in which one of the two alleles of the X chromosome is inactivated in female [1]. Recent studies have revealed that ASE is relatively common [2], and that many *cis*-acting sequence variants can alter gene expression in a highly context-specific manner [3]. In some cases, differences in the expression of two alleles can be predisposition to diseases, such as colorectal cancer [4]. Importantly, transcript abundances can be used as quantitative traits for identifying susceptibility loci for common diseases, such as diabetes and obesity [5, 6]. Hence, it is of our great interest to identify ASE and characterize genetic variants that are directly associated with phenotypic differences for elucidating causal mechanisms of diseases.

In order to identify allele-specific gene expression, RNA-sequencing (RNA-Seq) has now been widely used. However, there are several difficulties in measuring the amount of expressed isoforms in an allele-specific manner from RNA-Seq data given genotypes of an individual. First, in many cases, short reads can be aligned to multiple locations of the reference genome, which poses uncertainty in quantifying gene expression levels [7]. Statistical methods that handle ambiguous alignment of reads as hidden variables have been shown to be effective in optimizing read alignments for more accurate quantification of isoforms [8–10], although the approaches do not consider isoforms in an allele-specific manner. Another difficulty is that there is a bias in alignment of reads to the reference genome if a sample has heterozygous SNPs where nucleotides are different from the reference sequence [11–13]. To avoid the bias in alignment of reads to the reference genome, one can prepare the alternative allele that includes genomic variants [14, 15], or construct diploid genomes for a specific sample [16]. Then, the best alignments of reads to the extended reference sequences are used to count the number of the paternally or maternally derived reads based on heterozygous SNP sites. However, these approaches cannot quantify isoform expression levels accurately, since only reads that align heterozygous positions are considered for ASE. To our best knowledge, there is currently no approach that can estimate ASE explicitly as well as isoform abundances in a unified statistical framework, given RNA-Seq data and diploid genomes.

In this paper, we present a novel method called ASE-TIGAR, to estimate ASE as well as gene expression levels of isoforms simultaneously from RNA-Seq data and diploid genome sequences. In the read generative model, a haploid choice is modeled as a hidden variable, and the posterior distribution for the binomial random variable is estimated by variational Bayesian inference. In order to evaluate our approach, we prepare two sets of synthetic paired-end reads (30 million reads, 100 bp × 2) with some sequencing errors, one is generated based on the null-hypothesis where there is no ASE, and the other is generated based on the alternative hypothesis where there is ASE for a certain portion of isoforms. We apply ASE-TIGAR to the simulation data and show that our method identifies more ASE isoforms than those identified with the existing approach. We also show that our method predicts isoform abundances more accurately compared to TIGAR2, RSEM and Cufflinks, which are widely used software for isoform-level quantification from RNA-Seq data. Finally, we apply our method to the RNA-Seq data obtained from the human lymphoblastoid cell line GM12878 [17] to identify autosomal genes that exhibit ASE, and investigate the balance of X-chromosome inactivation between the paternal and maternal alleles in the cell line.

## Methods

### ASE-TIGAR pipeline

**Fig. 1:**
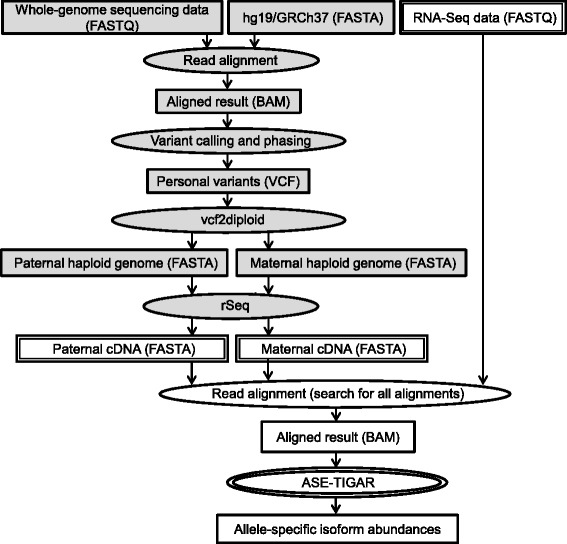
ASE-TIGAR pipeline for estimating ASE. The input data for ASE-TIGAR are RNA-Seq data, paternal and maternal cDNA sequences, represented as rectangles with double lines. Alternatively, whole-genome sequencing data can be used as an input with pre-processing steps (represented as shaded rectangles and circles)

A standard ASE-TIGAR pipeline starts from three input files, RNA-Seq data in FASTQ format, paternal and maternal cDNA sequences in FASTA format constructed from diploid genome sequences (represented as three rectangles with double lines in Fig. 1). In order to obtain cDNA sequences from the diploid genomes, “generate transcripts” function in rSeq software [18] can be used. Alternatively, it is also possible to construct diploid genome sequences from personal variants data in VCF format with vcf2diploid [16], or start from whole-genome sequencing data with the pre-processing steps described in Fig. 1 (shaded rectangles and circles). Then, RNA-Seq reads are aligned to the paternal and maternal cDNA sequences simultaneously, and all alignments are retained in BAM format. Bowtie2 [19] version 2.2.2 is used for searching all possible alignments for each read with “-k” option. Finally, ASE-TIGAR software takes the BAM file and estimate allele-specific isoform abundances after optimizing read alignments to the cDNA sequences of both alleles.

### Read generative model

**Fig. 2:**
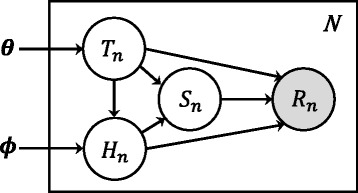
The read generative model in ASE-TIGAR. Model parameters, isoform abundances and allelic preferences are represented by ***θ*** and ***ϕ***, respectively. The isoform choice, haplotype choice, and nucleotide sequence of read *n* are represented by *T*_*n*_, *H*_*n*_, and *R*_*n*_, respectively

We use a graphical model, or Bayesian network, for representing a read generative model. For simplicity, here we describe a generative model for reads sequenced from single-end RNA-Seq libraries (Fig. 2). The model generates *N* independent and identically distributed reads, and each read *n* is associated with the three hidden variables *T*_*n*_,*H*_*n*_, and *S*_*n*_, and the random variable *R*_*n*_. The latent variable *T*_*n*_ represents the isoform choice of read *n*, and *T*_*n*_=*t* means that read *n* is generated from isoform *t*. The latent variable *H*_*n*_ represents the haplotype choice of read *n*, and *H*_*n*_=0 means that read *n* is generated from the paternal allele, whereas *H*_*n*_=1 means that read *n* is generated from the maternal allele. The latent variable *S*_*n*_ represents the start position of read *n*, and *S*_*n*_=*s* means that read *n* is generated from position *s* (1≤*s*≤*l*_*th*_−*L*+1), where *l*_*th*_ is the length of isoform *t* of haplotype *h* and *L* is the read length. The random variable *R*_*n*_ is the observed data and represents the nucleotide sequences of read *n*. There are two model parameter vectors, ***θ*** and ***ϕ***, which represent the isoform abundances and allelic preferences for isoforms, respectively. The parameter vector ***θ***=(*θ*_0_,…,*θ*_*T*_)^′^ represents the fraction of abundance for each isoform, where $\sum _{t=0}^{t=T} \theta _{t} = 1$. The parameter vector ***ϕ***=(*ϕ*_0_,…,*ϕ*_*T*_)^′^ represents the fraction of the paternal allele for each isoform, where 0≤*ϕ*_*t*_≤1.

As indicated in Fig. 2, conditional independence assumptions are used to simplify the model structure. Then, the complete likelihood of the data is decomposed as the product of conditional probabilities:

$$\begin{aligned} p\left(T_{n}, H_{n}, S_{n}, R_{n} \mid \boldsymbol{\theta}, \boldsymbol{\phi}\right) &= p\left(T_{n} \mid \boldsymbol{\theta}\right) p\left(H_{n} \mid T_{n}, \boldsymbol{\phi}\right)\\ &\quad\times p\left(S_{n} \mid T_{n},H_{n}\right)\\ &\quad\times p(R_{n} \mid T_{n},H_{n},S_{n}). \end{aligned} $$

*p*(*T*_*n*_=*t*∣***θ***) is the probability that read *n* is generated from isoform *t*, given ***θ***. We calculate this probability as *p*(*T*_*n*_=*t*∣***θ***)=*θ*_*t*_.

*p*(*H*_*n*_=*h*∣*T*_*n*_=*t*,***ϕ***) is the probability that read *n* is generated from haplotype *h* (either paternal or maternal), given the isoform choice and ***ϕ***. We calculate this probability as *p*(*H*_*n*_=0∣*T*_*n*_=*t*,***ϕ***)=*ϕ*_*t*_ (if read *n* is generated from the paternal allele), or *p*(*H*_*n*_=1∣*T*_*n*_=*t*,***ϕ***)=1−*ϕ*_*t*_ (if read *n* is generated from the maternal allele).

*p*(*S*_*n*_=*s*∣*T*_*n*_=*t,H*_*n*_=*h*) is the probability that read *n* is generated from position *s*, given isoform *t* and haplotype *h*. We calculate this probability as *p*(*S*_*n*_=*s*∣*T*_*n*_=*t,H*_*n*_=*h*)=1/(*l*_*th*_−*L*+1).

*p*(*R*_*n*_∣*T*_*n*_=*t,H*_*n*_=*h, S*_*n*_=*s*) is the probability of observing the nucleotide sequence of read *n*, given the isoform choice, haplotype choice, and start position of read *n*. To summarize hidden variables *T*_*n*_, *H*_*n*_, and *S*_*n*_, we introduce an indicator random variable *Z*_*nths*_, where *Z*_*nths*_ is equal to one if (*T*_*n*_,*H*_*n*_,*S*_*n*_)=(*t, h, s*) and zero otherwise. Let *π*_*n*_ be a set of all (*t, h, s*) tuples for possible alignments of read *n*. Then, for each (*t, h, s*)∈*π*_*n*_, we can calculate the probability of read sequence by

$$\begin{array}{@{}rcl@{}} p\left(R_{n} \mid Z_{nths}=1\right) = \prod_{x=1}^{L} subst\left(r_{n}[x], q_{n}[x], c_{th}[x]\right), \end{array} $$

where *subst*(·,·,·) is the quality score dependent substitution matrix [10], *r*_*n*_[*x*] is the nucleotide character of position *x* of read *n*, *q*_*n*_[*x*] is the quality score of position *x* of read *n*, and *c*_*th*_[*x*] is the nucleotide character of position *x* of cDNA sequence of isoform *t* of haplotype *h*. The quality score dependent substitution matrix, *subst*(·,·,·), is either determined according to the Phred base quality score [20], or can be estimated from the best alignments of reads to the reference cDNA sequences from the RNA-Seq data.

For the cases where RNA-Seq reads are generated from paired-end libraries, and how indel errors of sequencers can be handled, the previously proposed model [10] can be naturally extended similarly to the case for the single-end data described here.

### Variational Bayesian inference

We propose a Bayesian approach, in which model parameters are estimated as posterior distributions, given RNA-Seq data and prior distributions for the model parameters ***θ*** and ***ϕ***. Because full Bayesian inference involves integration over all possible hidden variable ***Z*** and is analytically intractable, we use variational Bayesian inference [21], which approximates the posterior joint distributions by assuming the factorization of latent variables and model parameters as *q*(***θ***,***ϕ***,***Z***)≈*q*(***θ***)*q*(***ϕ***)*q*(***Z***).

For the prior distribution of ***θ***, we use the Dirichlet distribution

$$\begin{array}{@{}rcl@{}} p\left(\boldsymbol{\theta}\right) = \frac{1}{G\left(\boldsymbol{\alpha}\right)} \prod_{t=0}^{T} \theta_{t}^{\alpha_{t} - 1}, \end{array} $$

where *α*_*t*_>0 is a hyperparameter, $G(\boldsymbol {\alpha }) = \frac {\prod _{t} \Gamma (\alpha _{t})}{\Gamma \left (\sum _{t} \alpha _{t}\right)}$, and *Γ*(·) is the gamma function. In this paper, we use a single hyperparameter *α*_0_ for all isoforms, based on the assumption that there is no prior knowledge about relative differences in isoform abundance. The single hyperparameter *α*_0_ controls the complexity of model parameters [22]. When *α*_0_≥1,*α*_0_−1 can be interpreted as the prior count of reads that are assigned to isoforms, and when *α*_0_<1, the prior favors that some of the isoform abundances to be zero [10]. Here, we choose *α*_0_ that maximizes the lower bound of the log marginal likelihood.

For the prior distribution of ***ϕ***, we use the Beta distribution

$$\begin{array}{@{}rcl@{}} p\left(\phi_{t}\right) = \frac{1}{B\left(\beta_{t,1}, \beta_{t,2}\right)} \phi_{t}^{\beta_{t,1} - 1} \left(1 - \phi_{t}\right)^{\beta_{t,2} - 1}, \end{array} $$

where *β*_*t*,1_>0 and *β*_*t*,2_>0 are hyperparameters, and *B*(·,·) is the Beta function. Here, *β*_*t*,1_ and *β*_*t*,2_ can be interpreted as the prior counts of reads that are assigned to the paternal and maternal allele, respectively, for calculating the paternal/maternal ratio. We use *β*_*t*,1_=*β*_*t*,2_=1 for all isoforms as a non-informative prior.

Given hyperparameters *α*_0_, *β*_*t*,1_, and *β*_*t*,2_, the lower bound of the log marginal likelihood is maximized iteratively by variational Bayesian inference algorithm:

- **Step 1**. Initialization
  For each isoform *t*, set $\alpha _{t}^{*} = \alpha _{0}$, $\beta _{t,1}^{*} = \beta _{t,1}$, and $\beta _{t,2}^{*} = \beta _{t,2}$
- **Step 2**. Update *q*^∗^(***Z***)
  Compute *E*_*Z*_[*Z*_*nths*_] given the current estimate of *q*^∗^(***θ***) and *q*^∗^(***ϕ***)
- **Step 3**. Update *q*^∗^(***θ***) and *q*^∗^(***ϕ***)
  Compute *E*_*θ*_[*θ*_*t*_] and *E*_*ϕ*_[*ϕ*_*t*_] given the current estimate of *q*^∗^(***Z***)
- **Step 4**. Check for convergence
  If none of the *E*_*θ*_[*θ*_*t*_] has been changed more than a pre-specified threshold, exit. Otherwise, return to Step 2

In Step 2, *E*_*Z*_[*Z*_*nths*_] is calculated based on the current estimate of *q*^∗^(***θ***) and *q*^∗^(***ϕ***) as

$$\begin{array}{@{}rcl@{}} E_{Z}\left[Z_{nths}\right] = \left\{ \begin{array}{ll} \frac{\rho_{nths}}{\sum_{(t^{\prime}h^{\prime}s^{\prime}) \in \pi_{n}} \rho_{nt^{\prime}h^{\prime}s^{\prime}}} &\text{if }(\text{\textit{t, h, s}}) \in \pi_{n}, \\ 0 \ \ \text{otherwise}. \end{array} \right. \end{array} $$

where

$$\begin{array}{@{}rcl@{}} \text{log} \rho_{nths} = \left\{ \begin{array}{l} E_{\theta}\left[\text{log} \theta_{t}\right] + E_{\phi}\left[\text{log} \phi_{t}\right] + \text{log}p\left(S_{n}|T_{n},H_{n}\right) \\ \quad+ \text{log}p(R_{n}|T_{n},H_{n},S_{n})\quad\text{if } h=0,\\ E_{\theta}\left[\text{log} \theta_{t}\right] + E_{\phi}\left[\text{log} (1 - \phi_{t})\right] \\\quad+ \log p(S_{n}|T_{n},H_{n})\\ \quad+ \log p(R_{n}|T_{n},H_{n},S_{n}) \text{ otherwise}. \\ \end{array} \right. \end{array} $$

Note that

$$\begin{array}{@{}rcl@{}} E_{\theta}\left[\text{log} \theta_{t}\right] = \psi\left(\alpha_{t}^{*}\right) - \psi\left(\sum_{t} \alpha_{t}^{*}\right),\\ E_{\phi}\left[\text{log} \phi_{t}\right] = \psi\left(\beta_{t,1}^{*}\right) - \psi\left(\beta_{t,1}^{*} + \beta_{t,2}^{*}\right),\\ E_{\phi}\left[\text{log} \left(1 - \phi_{t}\right)\right] = \psi\left(\beta_{t,2}^{*}\right) - \psi\left(\beta_{t,1}^{*} + \beta_{t,2}^{*}\right), \end{array} $$

where *ψ*(·) is the digamma function.

In Step 3, *E*_*θ*_[*θ*_*t*_] is calculated based on the current estimate of *q*^∗^(***Z***) as

$$\begin{array}{@{}rcl@{}} E_{\theta}\left[\theta_{t}\right] = \frac{\alpha^{*}_{t}}{\sum_{t^{\prime}} \alpha^{*}_{t^{\prime}}}, \end{array} $$

where

$$\begin{array}{@{}rcl@{}} \alpha^{*}_{t} = \alpha_{0} + \sum_{n^{\prime},t^{\prime}=\text{\textit{t,h}}^{\prime},s^{\prime}} E_{Z}\left[Z_{n^{\prime}t^{\prime}h^{\prime}s^{\prime}}\right]. \end{array} $$

Hence, it turns out that *q*^∗^(***θ***) is also the Dirichlet distribution, and the prior distribution *p*(***θ***) is the conjugate prior.

Similarly, *E*_*ϕ*_[*ϕ*_*t*_] is calculated based on the current estimate of *q*^∗^(***Z***) as

$$\begin{array}{@{}rcl@{}} E_{\phi}[\phi_{t}] = \frac{\beta_{t,1}^{*}}{\beta_{t,1}^{*} + \beta_{t,2}^{*}}, \end{array} $$

where

$$\begin{array}{@{}rcl@{}} \beta_{t,1}^{*} = \beta_{t,1} + \sum_{n^{\prime},t^{\prime}=\text{\textit{t,h}}^{\prime}=0,s^{\prime}} E_{Z}\left[Z_{n^{\prime}t^{\prime}h^{\prime}s^{\prime}}\right],\\ \beta_{t,2}^{*} = \beta_{t,2} + \sum_{n^{\prime},t^{\prime}=\text{\textit{t,h}}^{\prime}=1,s^{\prime}} E_{Z}\left[Z_{n^{\prime}t^{\prime}h^{\prime}s^{\prime}}\right]. \end{array} $$

Hence, *q*^∗^(*ϕ*_*t*_) is also the Beta distribution, and the prior distribution *p*(*ϕ*_*t*_) is the conjugate prior.

In Step 4, a relative change of 10^−3^ for isoforms whose abundance parameter *E*_*θ*_[*θ*_*t*_]>10^−7^ is used as a convergence criteria.

### Variational lower bound

The log marginal likelihood can be decomposed as

$$\begin{array}{@{}rcl@{}} \text{log} p(\boldsymbol{R}) = L(q) + KL(q||p), \end{array} $$

where

$$\begin{array}{@{}rcl@{}} L(q) = \int \int \int q\left(\boldsymbol{\theta}, \boldsymbol{\phi}, \boldsymbol{Z}\right) \text{log} \frac{p\left(\boldsymbol{R},\boldsymbol{\theta},\boldsymbol{\phi},\boldsymbol{Z}\right)}{q\left(\boldsymbol{\theta},\boldsymbol{\phi},\boldsymbol{Z}\right)} d\boldsymbol{\theta} d\boldsymbol{\phi} d\boldsymbol{Z}, \\  KL(q||p) = - \int \int \int q(\boldsymbol{\theta}, \boldsymbol{\phi}, \boldsymbol{Z}) \text{log} \frac{p\left(\boldsymbol{\theta},\boldsymbol{\phi},\boldsymbol{Z} | \boldsymbol{R}\right)}{q\left(\boldsymbol{\theta},\boldsymbol{\phi},\boldsymbol{Z}\right)} d\boldsymbol{\theta} d\boldsymbol{\phi} d\boldsymbol{Z}. \end{array} $$

Since *KL*(*q*||*p*) is the Kullback-Leibler divergence between *q*(***θ***,***ϕ***,***Z***) and *p*(***θ***,***ϕ***,***Z***|***R***), the log marginal likelihood is lower bounded by *L*(*q*). With the factorization assumption *q*(***θ***,***ϕ***,***Z***)≈*q*(***θ***)*q*(***ϕ***)*q*(***Z***), we have

$$\begin{array}{@{}rcl@{}} L(q) & = & E\left[\text{log}p\left(\boldsymbol{R},\boldsymbol{\theta},\boldsymbol{\phi},\boldsymbol{Z}\right)\right] - E\left[\text{log} q\left(\boldsymbol{\theta}, \boldsymbol{\phi}, \boldsymbol{Z}\right)\right] \\ & = & E\left[\text{log}p\left(\boldsymbol{R},\boldsymbol{Z} | \boldsymbol{\theta},\boldsymbol{\phi}\right)\right] + E\left[\text{\boldmath log} p(\boldsymbol{\theta})\right] + E\left[\text{\boldmath log} p(\boldsymbol{\phi})\right]\\ & & - E\left[\text{\boldmath log} q(\boldsymbol{\theta})\right] - E\left[\text{\boldmath log} q(\boldsymbol{\phi})\right] - E\left[\text{\boldmath log} q(\boldsymbol{Z})\right], \end{array} $$

where

$$\begin{array}{@{}rcl@{}} E\left[\text{log}p\left(\boldsymbol{R},\boldsymbol{Z} | \boldsymbol{\theta},\boldsymbol{\phi}\right)\right] & = & \sum_{n,t,h,s} E_{Z} [Z_{nths}] {\text{log} \rho_{nths}},\\ E\left[\text{log} p(\boldsymbol{\theta})\right] & = & \sum_{t} (\alpha_{0} - 1) E_{\theta} \left[\text{log} \theta_{t}\right] - \text{log} G(\boldsymbol{\alpha}),\\ E\left[\text{log} p(\boldsymbol{\phi})\right] & = & \sum_{t} \left\{ \left(\beta_{t,1} - 1\right)E_{\phi}\left[\text{log}\phi_{t}\right]\right. \\&& + \left.\left(\beta_{t,2} - 1\right)E_{\phi}\left[\text{log}\left(1 - \phi_{t}\right)\right] \right\}\\ & & - \sum_{t} \text{log} B\left(\beta_{t,1}, \beta_{t,2}\right),\\ E\left[\text{log} q\left(\boldsymbol{\theta}\right)\right] & = & \sum_{t}\ \left(\alpha_{t}^{*} - 1\right) E_{\theta}\! \left[\text{log} \theta_{t}\right] \,-\, \text{log} G\!\left(\boldsymbol{\alpha}^{*}\right),\\ E\left[\text{log} q\left(\boldsymbol{\phi}\right)\right] & = & \sum_{t} \left\{ \left(\beta_{t,1}^{*} - 1\right)E_{\phi}\left[\text{log}\phi_{t}\right]\right. \\&&+ \left.\left(\beta_{t,2}^{*} - 1\right)E_{\phi}\left[\text{log}\left(1 - \phi_{t}\right)\right] \right\}\\ & & - \sum_{t} \text{log} B\left(\beta_{t,1}^{*}, \beta_{t,2}^{*}\right), \\ E\left[\text{log} q(\boldsymbol{Z})\right] & = & \sum_{n,t,h,s} E_{Z} \left[Z_{nths}\right] {\text{log} E_{Z} \left[Z_{nths}\right]}. \end{array} $$

## Results and discussion

### Simulation data analysis

To evaluate the performance of the proposed method, we prepared synthetic RNA-Seq data (30 million reads, 100 bp × 2 with the mean fragment size of 400 bp and standard deviation of 40 bp) based on diploid genome sequences of NA12878, which were constructed from hg19 and publicly available from the website (http://sv.gersteinlab.org/NA12878_diploid). First, the paternal and maternal cDNA sequences were generated from the diploid genome sequences based on the UCSC gene annotations file (refFlat.txt) with rSeq (version 0.2.1) as described in Methods section. Second, 10,000 isoforms were randomly chosen and expression levels were assigned so that it follows the log-normal distribution. Then, we prepared two sets of RNA-Seq data with 0.1 *%* substitution, deletion, and insertion errors, one was generated based on the null hypothesis that there was no ASE, and the other was generated based on the alternative hypothesis that there were ASE for some portions of isoforms. For the null hypothesis data set, 100 *%* of the isoforms express the paternal and maternal alleles equally likely (50:50 chance). On the other hand, for the ASE data set, 10 *%* of the isoforms have the paternal-specific expression (in which the paternal allele was chosen to express with an 80 *%* probability, whereas the maternal allele was chosen to express with a 20 *%* probability), 10 *%* of the isoforms have the maternal-specific expression (in which the maternal allele was chosen to express with an 80 *%* probability, whereas the paternal allele was chosen to express with a 20 *%* probability), and the remaining isoforms have no ASE.

To compare with the existing approach [16], reads were aligned to the both paternal and maternal haplotypes, and the best alignments of reads were obtained. Then, for each isoform, the number of heterozygous SNPs was counted to determine the paternal/maternal ratio. On the other hand, our approach aligned reads to the both haplotypes and retained all the possible alignments with Bowtie2 specifying “-k” option. Then, ASE-TIGAR took the BAM file as input and optimized the read alignments between the paternal and maternal alleles, as well as among isoforms by variational Bayesian inference algorithm as described in Methods section. The hyperparameter *α*_0_ was set to 0.1, which maximized the variational lower bound of the marginal log likelihood of the data.

**Fig. 3:**
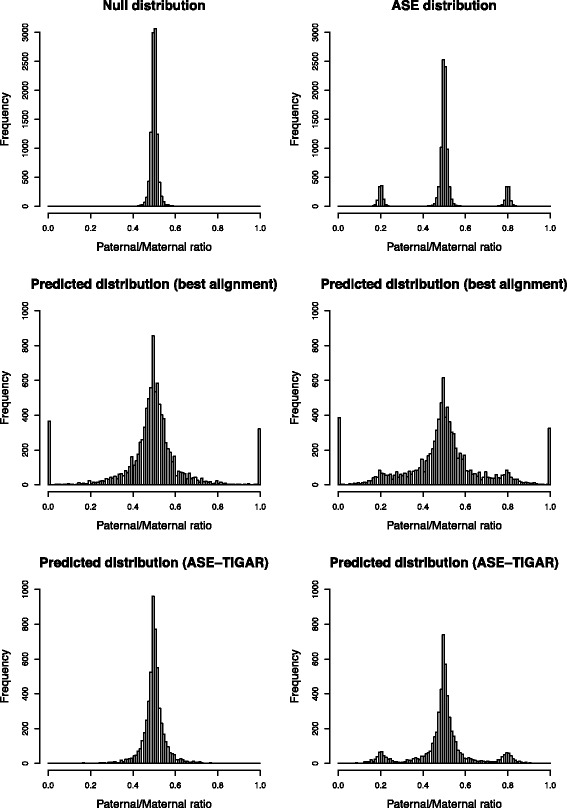
Estimation of ASE from simulated data. True distributions of the paternal/maternal ratio for the null and ASE hypotheses (*top-left* and *top-right*); predicted distributions with the existing approach for the null and ASE hypotheses (*middle-left* and *middle-right*); predicted distributions with ASE-TIGAR for the null and ASE hypotheses (*bottom-left* and *bottom-right*)

Predicted distributions of the paternal/maternal ratio for the null and ASE hypotheses with ASE-TIGAR and the existing approach (based on the best alignments of reads to the diploid genomes) are compared with the the true distributions (Fig. 3). Note that isoforms having one or more heterozygous SNP(s) with ten or more assigned reads were considered for the comparison. Whether there is ASE or not, the predicted distributions with ASE-TIGAR were more similar to the true distributions, particularly in the area where the paternal/maternal ratio is close to zero or one. On the contrary, the predicted distributions with the existing approach show “peaks” in those extreme area, which in fact did not exist in the true distributions. The favorable result with ASE-TIGAR came from the smoothing property of the updated beta distribution for the haplotype choice variable in the Bayesian inference, in which the prior count of one was naturally added to each allele of isoforms for calculating the paternal/maternal ratio (called as Laplace smoothing, or add-one smoothing). This feature will be especially beneficial for isoforms whose expression levels are inherently low, or when there are not many heterozygous SNPs that can be used to distinguish isoforms between paternal and maternal alleles.

**Fig. 4:**
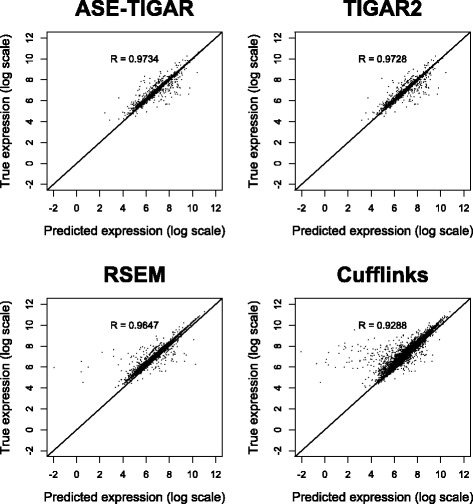
Estimation of isoform expression from simulated data. Scatter plots of isoform expression levels estimated from simulated RNA-Seq data (*x*-axis) and true expression levels (*y*-axis). The Pearson correlation coefficients are calculated and shown on each plot. Predictions with ASE-TIGAR were most consistent compared to TIGAR2, RSEM, and Cufflinks

Next, we evaluate the performance of quantifying isoform expression levels with ASE-TIGAR compared to existing methods using the simulation data. For comparing the performance, TIGAR2 [23], RSEM v1.2.21 [24] and Cufflinks v2.2.1 (with default options except ‘-u’ and ‘-G’ options) [25] are applied to the same simulation data. Note that TIGAR2, RSEM, and Cufflinks predict isoform expression levels without allelic information, and use the reference genome instead of the diploid sequences. Here, we compare the combined isoform expression levels (both paternal and maternal) predicted by ASE-TIGAR, with isoform levels predicted by TIGAR2, RSEM, and Cufflinks. The scatter-plot of the estimated isoform abundances (log of the number of reads) and the true isoform expression levels and the Pearson correlation are shown in Fig. 4. Root mean square errors were also calculated for comparison (ASE-TIGAR: 0.778, TIGAR2: 0.785, RSEM: 0.881, and Cufflinks: 1.26). The prediction accuracy with ASE-TIGAR compared to those with TIGAR2, RSEM and Cufflinks were found to be better, which proves the usefulness of ASE-TIGAR for quantifying isoform-level expression levels, in addition to identifying ASE.

### Real data analysis

We applied ASE-TIGAR to the RNA-Seq data (36.5 million reads of 100 bp × 2) that was generated from the lymphoblastoid cell line GM12878 [17], which is publicly available under the NCBI SRA accession number SRX245434. This cell line was derived from the HapMap NA12878 individual, whose diploid genomes were similarly obtained and used as in the simulation data analysis.

**Fig. 5:**
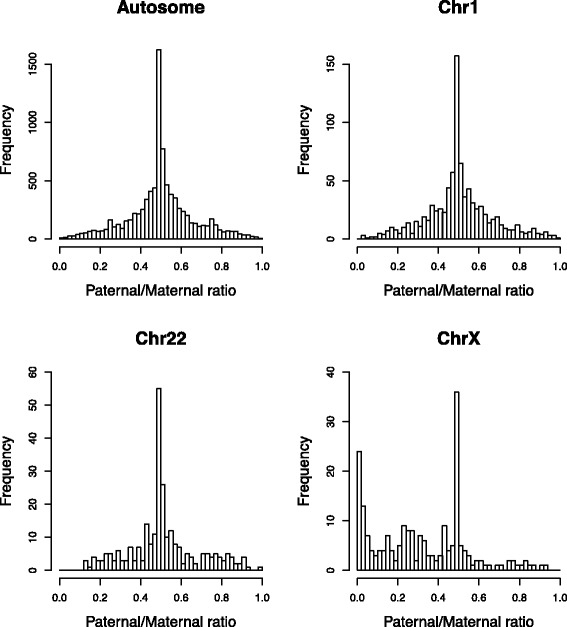
Estimation of ASE from GM12878 data. Estimated distributions for autosomal genes (*top-left*), genes on chr1 (*top-right*), genes on chr22 (*bottom-left*), and genes on chrX (*bottom-right*) with ASE-TIGAR

**Fig. 6:**
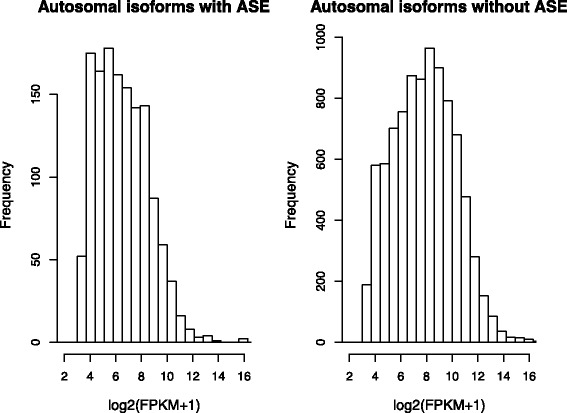
Estimation of autosomal isoform expression from GM12878 data. Frequencies of abundances of autosomal isoforms with ASE (*left*), and autosomal isoforms without ASE (*right*)

We found that there were some autosomal genes that showed ASE from either the paternal or maternal allele (top-left in Fig. 5). In the subsequent analysis, genes were considered as ASE genes, if the paternal/maternal ratio of their isoforms were either ≥0.75 or ≤0.25. To investigate which functional categories of genes were regulated in an allele-specific manner, we used DAVID [26] to identify enriched functional categories in the autosomal 1,251 ASE genes. Enriched terms included “polymorphism”, “sequence variant”, and “splicing variant” (Table 1), which might be explained by genomic variations among haplotypes within the population. For example, “polymorphism” annotation means that there is at least one variant within human, that is not directly responsible for a disease [27]. However, any functional category in the Gene Ontology Terms [28] was not found to be significant at the Bonferroni adjusted p-value of 0.001 in this analysis. When we compared overall abundances of autosomal ASE isoforms with those of autosomal isoforms without ASE, the former tend to be smaller than the latter (Fig. 6). This suggests that the lower expression from one allele due to genomic variants or other regulatory mechanisms were not compensated by the expression from the other allele in the cell line. Hence, genes showing ASE in the cell line were, in general, not likely to be house-keeping genes.

**Table 1:** Terms enriched in the autosomal ASE genes

Category	Term	Count	*P*-value	Bonferroni
SP_PIR	Polymorphism	787	9.7E-8	5.2E-5
UP_SEQ	Sequence variant	808	1.1E-7	3.2E-4
SP_PIR	Alternative splicing	599	1.6E-6	8.6E-4
SP_PIR	Glycoprotein	231	1.8E-6	9.7E-4
UP_SEQ	Extracellular	159	9.0E-7	2.7E-3
SP_PIR	Signal	170	1.0E-5	5.6E-3
UP_SEQ	Splice variant	597	2.4E-6	7.3E-3

SP_PIR: SwissProt Protein Information Resource Keyword. UP_SEQ: UniProt Sequence Feature

Interestingly, by looking at the paternal/maternal ratio of expressed isoforms on each chromosome, skewed X-inactivation in the paternal allele of the GM12878 cell line was observed (bottom-right in Fig. 5). This result is consistent with the findings in previous studies that showed the bias in X-chromosome inactivation by CTCF binding [29] and occupancies of RNA polymerase II [30] from ChIP-Seq data. ASE-TIGAR identified 90 maternal allele-specific isoforms on X-chromosome, whereas the existing approach based on the best alignment to the diploid genome [16] identified 76, based on the same experimental condition in the simulation analysis.

### Computational resources

Computational experiments were performed on a computer with Intel Xeon CPU E7-8837 processors (2.8 GHz) with the Red Hat Enterprise Linux Server release 6.1 operating system. ASE-TIGAR is implemented in Java and executed on 16 CPU cores with a multi-thread option. In the experiments for the simulated data sets (30 million paired-end reads), the execution time was 20 hours, and 46 GB memory was used with the Java(TM) SE Runtime Environment (build 1.8.0_45-b14).

## Conclusions

In this paper, we proposed a novel method called ASE-TIGAR, a Bayesian approach to estimate ASE from RNA-Seq data with diploid genomes. Contrary to the popularly used existing methods such as TopHat-Cufflinks [25], RSEM [8], and TIGAR2 [23], personal diploid genomes are used as reference sequences in the pipeline, instead of the reference genome. Since genetic variants such as SNPs and indels are incorporated in the diploid genome sequences by construction, there will be less bias in alignment of reads compared to the conventional approaches that rely on the reference genome. In the generative model, a haplotype choice is modeled as a latent variable and estimated simultaneously with isoform abundances by variational Bayesian inference.

We showed from the simulation data analysis that ASE-TIGAR estimated ASE more consistently compared to the existing approach, in part from smoothing effect of the estimated posterior distribution of the binomial random variable that represents the fraction of the expressed paternal and maternal haplotypes. We also showed that ASE-TIGAR quantified isoform abundances more accurately compared to TIGAR2, RSEM, and Cufflinks, which is an additional benefit of ASE-TIGAR if genotypes of samples are available. In the real data analysis of human lymphoblastoid cell line GM12878, ASE was identified among relatively low-expressed genes, and that no functional GO category was found to be significantly enriched. We also observed that the paternal X-chromosome inactivation was dominant in the cell line, which was also confirmed in the previous studies [29, 30].

Although full-length transcripts can be sequenced with new sequencing technologies, such as the PacBio RS II [31], accurate estimation of ASE is challenging without enough information about isoform abundances. Currently, the Illumina platform is more suitable in quantifying isoform abundances thanks to its capacity of generating short reads in a high-throughput manner. Because the accuracy of the reference sequences is critical for our approach, it will be effective to include the obtained full-length transcript sequences as reference cDNA sequences in ASE-TIGAR pipeline combined with short reads.

As more personal whole-genome sequencing data and RNA-Seq data become available [32], ASE-TIGAR will be particularly useful to find associations between genetic variants and expression quantitative loci (eQTL). For example, links between genetic variants in transcription factor (TF) binding sites and the level of gene expression can be investigated. Incorporation of other omics data, such as ChIP-Seq data measuring CTCF binding, TF occupancies, histone modifications, or chromatin structures will be possible in the similar framework. If only a limited portion of genotypes is available for samples (such as with SNP arrays), genotype imputation with the reference panel can be considered [33]. However, there might exist imputation errors, or switching errors in phased genotypes without a complete parental genotypes, which will affect accuracies in ASE identification and isoform quantification with ASE-TIGAR. Our future work will include investigating ASE with other cell types, and the topics described above.
